# AIDS, conflict and the media in Africa: risks in reporting bad data badly

**DOI:** 10.1186/1742-7622-2-12

**Published:** 2005-12-13

**Authors:** Massimo Lowicki-Zucca, Paul Spiegel, Filippo Ciantia

**Affiliations:** 1 Child Protection Section, UNICEF, New York, USA; 2 Division of Operational Support, UNHCR, Geneva, Switzerland; 3 Associazione Volontari per il Servizio Internazionale, Kampala, Uganda

## Abstract

**Background:**

Conflict, poverty and HIV disproportionately affect people in sub-Saharan Africa. The manner in which governments, national and international organisations and the media report on the HIV epidemic in situations of conflict, post-conflict and reconstruction can have unintended and negative consequences for those affected populations. The media in particular has a huge influence on how the world observes and reacts to the HIV epidemic among conflict-affected and displaced populations.

**Discussion:**

Three case studies focused on Sudan, Uganda and Guinea describe what the media reported and why the reports were incomplete, misleading or incorrect. The exploration of possible ways to ensure that the media do not unwittingly inflame delicate and complicated situations of HIV among conflict-affected and displaced populations is then undertaken using epidemiological and journalistic principles. The discussion is divided into four sections: 1) Avoid stigmatising statements and ensure a balanced view; 2) Avoid accurate but misleading statements; 3) Avoid inaccurate statements by clearly stating sources and verifying their credibility; and 4) Do not repeat data and conclusions from other news sources without checking their accuracy. The aim of this manuscript is to stimulate awareness and debate among persons and organisations working on HIV/AIDS as well as the media in order to improve dialogue and ultimately to reduce stigma and discrimination amongst an already vulnerable group – conflict-affected and displaced persons.

**Summary:**

The media and humanitarian organisations have published misleading and inaccurate HIV data and statements on conflict-affected and displaced populations in Sudan, Uganda and Guinea. Given the unique characteristics of the HIV epidemic and conflict-affected and displaced populations, the media have a special obligation to report in a balanced and non-discriminatory manner that may go beyond the accepted standards of journalism. The media may wish to have the HIV data and their interpretation reviewed by technical experts before going to press. Specific training for reporters and editors regarding ethical issues and basic epidemiological methods may help them to better understand the complexity of the situation and report more accurately; similar training for media watch groups and human rights organisations may improve the monitoring of such situations and possibly reduce misreporting and subsequent discrimination. More rigorous HIV biological and behavioural surveillance should be undertaken in situations of conflict and displacement and humanitarian guidelines should be integrated with guidance on media relations and reporting responsibilities of humanitarian agencies. Finally, humanitarian agencies must ensure the data they release are sound and that any biases are clearly stated. Improved communication with the media will help to ensure more accurate reporting and interpretation.

## Background

Conflict, poverty and HIV disproportionately affect people in sub-Saharan Africa. In 2004, 25 active armed conflicts occurred worldwide, 69 countries were affected by refugee movements and 48 countries by internal displacement of their populations [1]. Africa was the most affected continent. The current estimated number of refugees and internally displaced persons (IDPs) worldwide are 12 and 25 million, respectively [2,3]. Their needs are enormous due to the trauma they have suffered and the deplorable conditions in which they live. Furthermore, their vulnerabilities are increased due to destitution, displacement, discrimination and reduction of basic services and coping mechanisms. Significant challenges lie ahead for those who have become displaced, living in new and sometimes hostile environments. Repatriation to their country of origin also poses new challenges as well as problems.

Perceived HIV status, regardless of one's actual status, carries with it a stigma that can destroy individuals, their families and even communities. The manner in which governments, national and international organisations and the media report on the HIV epidemic in situations of conflict, post-conflict and reconstruction can have unintended and negative consequences for those affected populations including the aggravation of xenophobic fears, the imposition of mandatory HIV testing by authorities to regulate immigration or refugee inflows, refoulement (forced return of refugees to their country of origin), the hampering of reconciliation processes and creating obstacles to their integration in host communities and repatriation to countries of origin.

Everywhere in the world, people are very sensitive to the fears of disease being "brought in" by outsiders. Their fears can be exploited by some and used to promote political agendas. A recent example is the measures proposed by the United Kingdom's Conservative Party, after intense public and political debate about the scale and nature of asylum and migration, to affirm that "the [HIV] risk to the population comes primarily from those seeking permanent residency in the U.K, either as an asylum seeker or through the immigration service" [4]. Such arguments have been countered elsewhere, on the basis of limited evidence as well as practical and ethical problems [5,6].

The media has a huge influence on how the world observes and reacts to the HIV epidemic among conflict-affected and displaced populations. Therefore, as already argued for health and medical reporting in general [7,8], the media have a special responsibility to report on this sensitive topic knowledgeably and prudently, to consider and report on all sides of this complex issue, and to take into account any harmful implications their reporting may have for such populations [9].

The case studies in three conflict-affected African countries presented below state what the media reported and why the reports were incomplete, misleading or incorrect. The exploration of possible ways to ensure that the media do not unwittingly inflame delicate and complicated situations of HIV among conflict-affected and displaced populations by reporting and repeating misleading or incorrect statements from individuals, organisations and media sources is then undertaken. This article is meant to stimulate awareness and debate among persons and organisations working on HIV/AIDS as well as the media in order to improve dialogue and ultimately to reduce stigma and discrimination against an already vulnerable group – conflict-affected and displaced persons.

### Sudan

*"The fact that many Sudanese will return to their homes from countries where the HIV/AIDS prevalence is very high, will doubtless increase the likelihood of a further spread of the epidemic" *[10].

Conflict in Sudan has displaced millions of people and forced hundreds of thousands to flee across borders. In the south of the country, the 21-year old war between the Government of Sudan and the Sudan People Liberation Movement/Army has caused the internal displacement of approximately 4 million people, to which the more than 1.5 million displaced as a consequence of the more recent conflict in Darfur must be added. As of the end of 2003, more than 500,000 Sudanese refugees were residing mainly in Uganda, Ethiopia, Kenya and Chad [11].

By the end of 2003, the overall HIV prevalence among adults in Sudan was estimated at 2.3% (95% confidence interval (CI) 0.7 to 7.2) [12]. In 2002, the Sudanese government undertook an HIV survey in 14 of 26 states with the following results: pregnant women at antenatal clinics (1.0%), tuberculosis (TB) patients (1.6%), sexually transmitted infections (STI) patients (1.1%), sex workers (4.4%), tea sellers (2.5%), IDPs (1.0%) and Eritrean refugees (4.0%) [13].

Following progress in the negotiations between the Government of Sudan and the Sudan People Liberation Movement/Army, future peace in South Sudan and the consequent return home of refugees and IDPs was reported widely in the media. Particular attention was expressed regarding high infection rates among Sudanese IDPs and refugees, and the fear that refugees living in host countries with high prevalence rates will return and exacerbate the epidemic in South Sudan [14,15]. The situation was described as "severe", "on the verge of an HIV/AIDS epidemic" and a "disaster" that was going to "doubtless increase the likelihood of a further spread of the epidemic" [10,15,16].

### Uganda

Headline: *"HIV/AIDS soars in war-torn northern Uganda" *[17].

Northern Uganda is currently devastated by a 19-year old conflict between the Ugandan Government and the Lord's Resistance Army (LRA), which escalated in 1996 causing massive population displacement, further aggravated since the increase of violence in mid-2002. At the end of September 2004, there were 1.4 million IDPs in some 200 camps, living in extremely difficult hygienic and environmental conditions and depending on relief food distribution [18].

Uganda experienced declines in HIV prevalence during the 1990s. National prevalence estimated by antenatal sentinel surveillance peaked in the early 1990s and has fallen to 4.1% (95% CI 2.8 to 6.6) by the end of 2003 [12]. Sentinel surveillance shows that median HIV prevalence in the capital Kampala declined from a high of 29.4% in 1992 down to 8.3% in 2002, while in areas outside major urban centres it decreased from 12.0% to 4.6% [19].

Several reports in the national and international press and information disseminated by international organizations have emphasized that the HIV prevalence in northern Uganda (11.9% in 2002) is nearly twice as high as the national rate (6.2% in 2002) and growing due to conflict [20]. These reports particularly highlighted the high HIV prevalence among IDPs in the camps and the highest prevalence among victims of the LRA [21-26]. Children, particularly girls, captured by the LRA have been reported to have an HIV prevalence of approximately 50% [17,23,27-29]. In the past, this vulnerable group had already been suggested to be infected with extremely high rates of STIs, including HIV [30]. Furthermore, direct reference to "immorality" among IDPs as explanation for the higher rates of HIV prevalence was attributed to unspecified "NGO (non-governmental organisation) workers" [31].

### Guinea

*"It's the refugees' fault. AIDS comes with them!" *[32].

Since 1990, the republic of Guinea with a population of 7.5 million persons has hosted hundreds of thousands of refugees from primarily Sierra Leone and Liberia. Until late 2000, the refugees lived in small villages scattered throughout the rural south-eastern Forest region. During September to December 2000, attacks by armed factions in Guinea led to the displacement and consequent transfer of the refugees to safer camps in the north-west of the country [32]. By the end of 2004, there were still approximately 150,000 refugees primarily from Liberia, Sierra Leone and Cote d'Ivoire [2]. Due to recent conflict in Cote d'Ivoire, which has the highest HIV prevalence in the West Africa (7.0%) [12] over 100,000 Guineans have returned home, many to the Forest Region [32].

A national HIV seroprevalence survey, undertaken in Guinea in December 2001, showed an overall HIV prevalence of 2.8% among pregnant women with 4.4% in urban areas and 2.2% in rural areas [33,34]. An HIV prevalence of 7.0% was reported in two towns in the Forest Region, the area where the refugees were located. By the end of 2003, HIV prevalence was estimated at 3.2% [12].

Before the national survey, it was assumed that the overall HIV prevalence in Guinea was lower than 2.8%. The media reported that the higher HIV prevalence in the Forest Region was likely due to the influx of refugees, blaming their "loose morals" that resulted in "flourishing promiscuity and prostitution" [32].

## Discussion

The International Federation of Journalists' Declaration of Principles on the Conduct of Journalists is proclaimed as a standard of professional conduct for journalists engaged in gathering, transmitting, disseminating and commenting on news and information in describing events [35]. Of the eight principles stated, two are particularly applicable for this article:

I. The journalist shall report only in accordance with facts of which he/she knows the origin. The journalist shall not suppress essential information or falsify documents.

II. The journalist shall be alert to the danger of discrimination being furthered by media, and shall do the utmost to avoid facilitating such discriminations based on, among other things, race, sex, sexual orientation, language, religion, political or other opinions, and national and social origins.

We discuss below the critical aspects of the coverage of these three case studies by media reports following four main arguments:

### 1. Avoid stigmatising statements and ensure a balanced view

*"The fact that many Sudanese will return to their homes from countries where the HIV/AIDS prevalence is very high will doubtless increase the likelihood of a further spread of the epidemic" *[10].

*"Infection rates are particularly high among vulnerable groups, such as internally displaced persons (IDPs) and refugees" *[10].

Concern regarding stigmatizing statements against refugees living in Sudan and Sudanese refugees living in surrounding host countries began after the Integrated Regional Information Network (IRIN) [10] reported on a UN newsletter [16]. Although refugees often come from countries of origin with lower HIV prevalence and move to live among host country populations with higher HIV prevalence, each case is context specific and must be examined independently [36]. The degree of interaction among refugees and host communities as well as their HIV knowledge and behaviour all contribute to the HIV prevalence in both populations. Sudanese refugees living in Kenya and Uganda still have much lower HIV prevalence than their surrounding host communities despite living among them for over a decade [36]. Furthermore, Sudanese refugees living in Kenya have shown better knowledge and less risky behaviour than some Sudanese living in South Sudan [37]. Blanket statements such as the first one in the text box above are unhelpful and can be damaging. These and other statements in the UN report and IRIN article were published without checking with other sources, such as the United Nations High Commissioner for Refugees, NGOs working with the refugees, or the refugees themselves. After discussion with IRIN, the news organization acted in a rapid and professional manner. They removed the original article from their website, interviewed different experts in the field to provide a more balanced view, and changed the first statement listed above to "The fact that many Sudanese will return to their homes from countries where the HIV/AIDS rate are high *might *increase the likelihood of a further spread of the epidemic" [15].

*Headline: "GUINEA: Refugee influx adds fuel to AIDS crisis in southeast Guinea" *[32].

Another IRIN report stated that refugees are being blamed for spreading HIV among the local Guinean population. Statements from local Guineans and even refugees provide credence to the assertion that refugees spread HIV. However, the HIV prevalence has never been measured among refugees in Guinea and there is insufficient evidence to know the effect that the mixing of refugees and locals have had on the HIV prevalence in the region. Only near the end of the article does it state that condom use and knowledge of HIV/AIDS were much lower among Guinean youth than Liberian refugees.

### 2. Avoid accurate but misleading statements

*"The HIV-infection rate in Sudan, according to UNFPA, is already considered an epidemic, making Sudan the country with the highest infection rate in North Africa and the Middle East region" *[10].

Although Sudan has the highest HIV prevalence in North Africa and the Middle East, it has the lowest prevalence compared with the countries surrounding it to the East, West and South [12]. The former statement was made to buttress the point that Sudan has a serious HIV problem. Indeed, HIV is a very serious issue in Sudan; however, the fact remains that HIV prevalence is much higher in surrounding countries than Sudan itself.

*"The rate of HIV/AIDS infection in northern Uganda is nearly double that in the rest of the country..." *[29].

HIV prevalence in the Northern Uganda, represented by the surveillance site located in Lacor hospital in Gulu, is often correctly reported as nearly double the national average. This point estimate is then used to 'prove' that the conflict in Northern Uganda has caused the HIV epidemic to flourish. The estimated HIV prevalence for Gulu is however not the only one above the national average and is in fact similar to other urban sentinel sites in areas not affected by conflict (Table 1). Moreover, trends over time actually show that the HIV prevalence in Northern Uganda has decreased over time, similar to many other sentinel surveillance sites in the rest of the country. The decrease in the HIV prevalence in Gulu has been significant from 27% in 1993 to 12.8% in 1998 to 11.9% in 2002 (p < 0.001) [19,38]. The trends in Table 1 clearly show that, from 1993 to 2002, the reduction of HIV prevalence in the sentinel site for Northern Uganda has been larger than that of many other sentinel sites. Despite an increase in the intensity of the conflict from 1996 onwards, trends from 1998 to 2002 show a larger decrease in Gulu than in peaceful southern and eastern Uganda, represented by the sentinel site of Mbarara and Mbale regional hospitals, respectively.

**Table 1:** Decline in HIV prevalence rates, 1993–2002. Selected sentinel sites, Uganda.

			**HIV Prevalence**				
**Site**	**District**	Urban/ rural*	**1993**	**1998**	**2002**	**% change (2002/1993)**	**% change (2002/1998)**

Nsambya	Kampala TC	Urban	26.6	13.4	8.5	50.4%	63.4%
Rubaga	Kampala TC	Urban	24.4	14.2	8.1	58.2%	57.0%
**Mbarara**	**Mbarara**	**Urban**	**18.1**	**10.9**	**10.8**	**60.2%**	**99.1%**
Jinja	Jinja	Urban	16.7	10.5	5	62.9%	47.6%
Tororo	Tororo	Rural	11.3	10.5	6.3	92.9%	60.0%
**Mbale**	**Mbale**	**Urban**	**8.7**	**6.3**	**5.9**	**72.4%**	**93.7%**
Soroti	Soroti	Rural	9.1	7.7	4.6	84.6%	59.7%
Matany	Moroto	Rural	2.8	1.3	0.7	46.4%	53.8%
Mutolere	Kisoro	Rural	4.2	2.5	1.5	59.5%	60.0%
**Gulu**	**Gulu**	**Urban**	**27.0**	**12.8**	**11.9**	**47.4%**	**93.0%**

* Urban population defined as >50,000 persons and rural population as <50,000 persons Source: adapted from [19]

Therefore, basic epidemiological principles must be used when interpreting HIV prevalence data. Trends over time must be examined as opposed to comparing point prevalence estimates as occurred above.

### 3. Avoid inaccurate statements by clearly stating sources and verifying their credibility

*"Data from the mid-1990s onwards suggested the infection rates had risen rapidly in the conflict-affected areas of southern and western Sudan. According to a national prevalence and behaviour survey conducted in 2002, HIV prevalence was already four percent among pregnant women attending clinics in the refugee camps" *[10].

There are no data at present suggesting that the HIV prevalence in South and West Sudan is rising rapidly. No source is provided for the statement in the above text box. In South Sudan, IRIN reported that the Sudan Evangelical Mission found HIV prevalence to be 4–10% in Rumbeck and 17–21% in Yambio in their voluntary counselling and testing (VCT) centres [10]. However, VCT data present biases and cannot be used as proxy for HIV prevalence in the general population due to the biases involved in self selection of clients and referrals from medical personnel. As a comparison, HIV prevalence rates from VCT services in both Kitgum and Pader districts in neighbouring north Uganda, are estimated at 20% (AVSI-supported PMTCT sites program data. unpublished), whereas PMTCT results show the HIV prevalence to be 7.9%–9.9% in Kitgum and 4.6% in Pader [39].

The second sentence in the above quotation discusses HIV prevalence among Eritrean refugees living in the Eastern part of the country from the Sudanese National AIDS Control Programme's 2002 report. The comparison, however, should not be among Sudanese pregnant women attending antenatal care (ANC) from all over the country (HIV prevalence of 1.0%) with refugee pregnant women in East Sudan (4.1%) but rather between Sudanese pregnant women from the sentinel site nearest to the refugee camps (El Gedarif). The data provided by the 2002 report does not allow disaggregation by state. However, in 1998, Sudan reported the HIV prevalence in the ANC sentinel surveillance in El Gedarif as 4.0% [37,40], almost identical to the 4.1% reported among the refugees. There is limited HIV data from Eritrea; one sentinel site outside an urban area in 1994 showed 3% prevalence while one major urban area site in 1999 showed 4%; both are similar to the sites found in El Gedarif in Sudan and among the refugees [41]. Finally, the same IRIN article also claims that HIV infection rates are particularly high among vulnerable groups, such as IDPs and refugees. However, this is also incorrect; the HIV prevalence among IDPs was estimated to be 1.0% [13,36].

*"About half the girls who escape from the rebels are found to be HIV positive, doctors say" *[25].

Claims of extremely high HIV prevalence among children abducted by the LRA are not supported by data; no systematic HIV testing has ever been undertaken among this group. The statement reported above, originally made by a Government official [27] and then reported by numerous NGOs and news agencies, was subsequently dismissed by organizations responsible for the major reception centres for these children in the region (personal communications and email exchanges between members of the Psychosocial Working Group and Child Care Centres in North Uganda, August 2004).

### 4. Do not continuously repeat data and conclusions from other news sources without checking for accuracy

*[AIDS is the] "leading cause for death, constituting 69% of deaths in Gulu area, or three times higher than direct killings during military confrontation" *[26].

**Figure 1:**
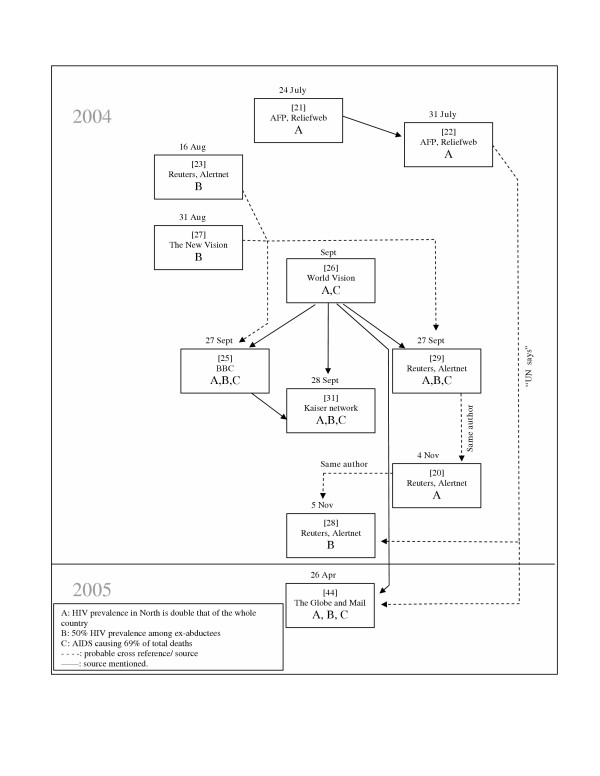
Sequence of reporting of three main incorrect statements on HIV and North-Uganda.

Misleading or false information regarding HIV and AIDS in Northern Uganda has been parroted repeatedly by news agencies, apparently without verification. Figure 1 attempts to trace the origin and the subsequent reporting between July and November 2004 of three main statements: 1) HIV prevalence in Northern Uganda is double that of the whole country; 2) HIV prevalence is 50% among those children abducted and subsequently released by the LRA; and 3) AIDS is the cause of 69% of the total deaths in Northern Uganda.

The first two issues were discussed previously and have been shown to be incorrect. The third issue relates to a recent report on the humanitarian situation of Northern Uganda which claimed AIDS to be the leading cause of death (69%) in the Gulu area, three times higher than direct killings during military confrontation [26]. The source for this statement was not provided, and coverage of HIV testing is limited to few areas within Gulu District. Conversely, Médecins Sans Frontières undertook at the end of 2004 a population-based survey and reported alarmingly high rates of mortality in the insurgent-affected districts of Lira and Pader in Northern Uganda (crude mortality rate of 2.8/10,000/day and under five mortality rate of 5.4/10,000/day) [42]. As in the Democratic Republic of Congo [43], the main causes of death were indigenous communicable diseases that have been exacerbated due to the conflict, such as malaria (47% of all deaths), respiratory disease (28%) and diarrhoeal disease (21%); however, AIDS could be an underlying factor that was not reported.

To further support this point, several months after the initial and incorrect statements were reported, all three re-emerged in the media [44] and, more recently, IDPs in North Uganda have been reported as having an average HIV/AIDS prevalence six times higher than the national average [45].

## Summary

The relationship between HIV/AIDS and conflict-affected and displaced populations is complicated and is influenced by numerous complex and competing factors, including the HIV prevalence in the area of origin and host communities, the amount of time since displacement, and the interaction between the affected and host communities [36,46,47].

This complex relationship is not unidirectional; conflict and displacement does not necessarily translate into higher HIV infection rates among affected persons. However, these situations increase the vulnerability of all persons, affected and host populations, to HIV infection. The incorrect, incomplete or misleading reporting of data and statements does not help to inform HIV policy or programming; rather it may reinforce discriminatory attitudes that stigmatise a population in need of protection and support.

Whilst misinformation in the media may be due to poor understanding and limited interest about disease patterns and measurement, it may also be due to the preference of the media for reporting dramatic stories. This is exemplified by the frequently negative portrayal of Africa by the international press. As a result, basic training for journalists in epidemiology may not be sufficient and alternative channels would need to be explored such as training of media watch groups and human rights organisations.

Given the unique characteristics of the HIV epidemic and conflict-affected and displaced populations, the media have a special obligation to report on this disease in a balanced and non-discriminatory manner that may go beyond the accepted standards of verifying sources. Many of the articles and reports cited in this paper did not provide their sources, the methodologies of collecting the data, or its possible biases.

To ameliorate the situation and avoid such situations in the future, we recommend that the media have HIV data and their interpretation reviewed by technical experts before going to press. Specific training for reporters and editors regarding ethical issues and basic epidemiological methods may help them to better understand the complexity of the situation and report in a more accurate manner. Similar training for media watch groups and human rights organisations may improve the monitoring of such situations and possibly reduce misreporting and subsequent discrimination. More rigorous HIV biological and behavioural surveillance surveys must occur in situations of conflict and displacement as this will provide data for decision makers and help to reduce the likelihood of incorrect, incomplete or misleading statements. The assertion that rigorous data collection is not possible during the acute phase of a crisis or immediately post-conflict is incorrect, as shown by several surveys implemented in such situations: DRC [43], Iraq [48], Kosovo [49], Sudan [50] and Uganda [42]. Humanitarian guidelines should be updated to include details on media relations and responsibilities of humanitarian agencies for reporting on mortality and morbidity; such issues are not included in either the Sphere Project containing the humanitarian charter and minimum standards on disaster response [51] or in the IASC Guidelines for HIV/AIDS Interventions in Emergency Settings [46]. Finally, humanitarian agencies must ensure the data they release are sound and biases are clearly stated. Improved communication with the media will help to ensure more accurate reporting and interpretation.

## Abbreviations

AIDS: Acquired Immunodeficiency Syndrome

ANC: Antenatal Care

AVSI: Associazione Volontari per il Servizio Internazionale

DRC: Democratic Republic of Congo

HIV: Human Immunodeficiency Virus

IDPs: Internally Displaced Persons

IRIN: Integrated Regional Information Network

LRA: Lord's Resistance Army

MSF: Médecins Sans Frontières

NGO: Non-Governmental Organisation

PMTCT: Prevention of Mother-to-Child Transmission (of HIV)

SNAP: Sudanese National AIDS Control Program

STIs: Sexually Transmitted Infections

VCT: Voluntary Counselling and Testing

## Competing interests statement

The authors and their institutions have no financial or other conflicts of interests.

There were no grants or outside funding for this work.

## Authors' contributions

Author 1 (MLZ) wrote the paper and undertook the literature review.

Author 2 (PS) conceived of the concept, designed the outline, and co-wrote the paper.

Author 3 (FC) participated in the literature review and co-wrote the paper.
